# Myoid hamartoma of the breast that proved difficult to diagnose: a case report

**DOI:** 10.1186/1477-7819-10-12

**Published:** 2012-01-16

**Authors:** Naruhiko Mizuta, Koichi Sakaguchi, Mitsuhiko Mizuta, Aya Imai, Katsuhiko Nakatsukasa, Midori Morita, Mari Soshi, Mariko Goto, Satoru Yasukawa, Eiichi Konishi, Tetsuya Taguchi

**Affiliations:** 1 Department of Endocrine and Breast Surgery, Kyoto Prefectural University of Medicine, Kawaramachi, Hirokoji, Kamikyo-ku, Kyoto 602-0841, Japan; 2 Department of Anesthesiology, Kyoto Prefectural University of Medicine, Kawaramachi, Hirokoji, Kamikyo-ku, Kyoto 602-0841, Japan; 3 Department of Radiology, Kyoto Prefectural University of Medicine, Kawaramachi, Hirokoji, Kamikyo-ku, Kyoto 602-0841, Japan; 4 Department of Pathology, Kyoto Prefectural University of Medicine, Kawaramachi, Hirokoji, Kamikyo-ku, Kyoto 602-0841, Japan

**Keywords:** myoid hamartoma, breast tumor, smooth-muscle actin

## Abstract

Myoid hamartomas of the breast are extremely rare breast lesions, with a poorly understood pathogenesis. We describe the case of a 38-year-old premenopausal woman who presenting with a mass in the left breast. Mammography revealed an oval mass that was partly indistinct, and ultrasonography showed a hypoechoic mass with a slightly irregular margin. Bilateral breast dynamic magnetic resonance imaging was performed for a more detailed evaluation. The images showed rapid initial enhancement and a microlobulated margin. Because the suspicion of malignancy was strong at that time, core needle biopsy was performed. Histologically, the tumor was identified as fibroadenoma. A case of myoid hamartoma of the breast that proved difficult to diagnose is reported, and discussed with reference to the literature.

## Background

Hamartoma is an uncommon breast tumor that histologically contains lobular breast tissue with various admixtures of fibrous, fibrocystic, and adipose tissue [1,2]. Myoid hamartoma is an extremely rare subtype containing an additional smooth muscle component [3]. Both radiographic correlations and immunohistochemical studies are important to diagnose myoid hamartoma and avoid confusion with other breast diseases. Herein, we report a case of myoid hamartoma of the breast that proved difficult to diagnoses because the findings on morphological evaluation employing various imaging modalities were different from the histological findings of core needle biopsy.

## Case presentation

**Figure 1:**
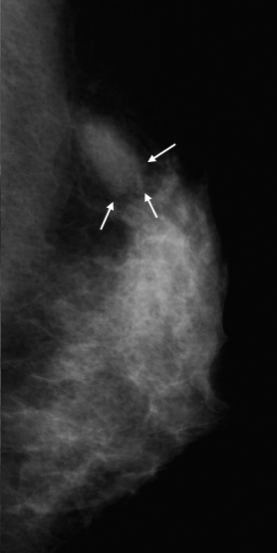
**Mammography of the left breast, showing a 1.1 × 2.2-cm, well-demarcated, oval, isodense mass that was partly indistinct (arrow)**.

**Figure 2:**
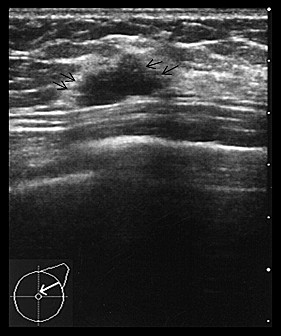
**Ultrasonography of the breast shows a 20 × 15 × 8.8-mm, well-demarcated, low-echoic lesion with slightly irregular margins (arrow)**.

**Figure 3:**
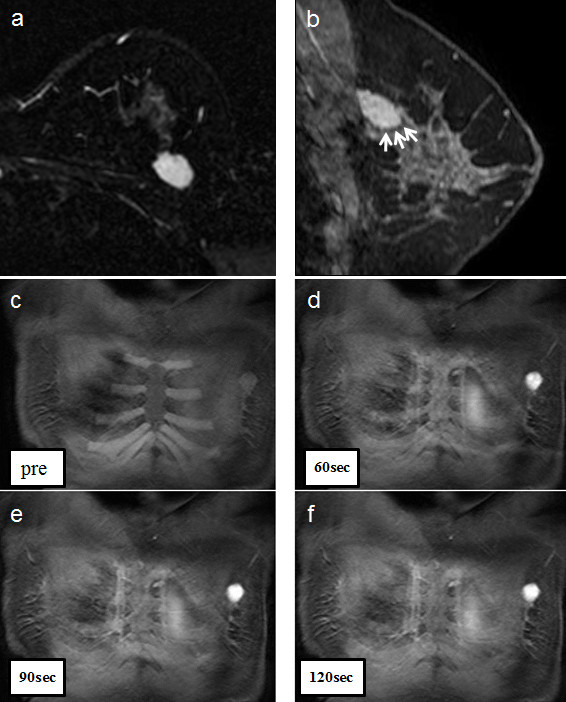
**a) MRI of the left breast, showing a well-circumscribed mass with a high signal intensity on T2WI**. b) On contrast-enhanced T1WI, the left breast lesion appeared as an enhanced mass with a microlobulated margin. c-f) The lesion on dynamic contrast MRI shows a peak in signal intensity within 90 seconds, after contrast injection.

**Figure 4:**
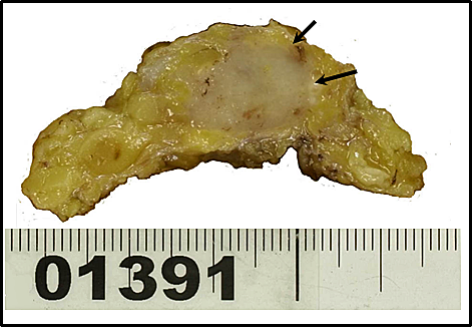
**Gross pathologic appearance of the myoid hamartoma**. The cut surface of the lesion shows a yellow-white, well-circumscribed, non-encapsulated mass, resembling a fibroadenoma.

A 38-year-old, premenopausal woman was admitted with a mass of the left breast. Family and past medical histories were unremarkable. Physical examination revealed a 19 × 28-mm mass in the upper outer quadrant of the left breast. The tumor was mobile and not fixed to the chest wall. The lymph nodes were not palpable in the left axilla, and no mass was present in the right breast. The patient was referred for mammography and ultrasonography. Mammography showed a 22-mm, well-demarcated, oval, isodense mass that was partly indistinct (BI-RADS category 4) (Figure 1), and ultrasonography revealed a 20 × 15 × 8.8-mm, well-demarcated, hypoechoic lesion with slightly irregular margins in the outer part of the left breast (Figure 2). Because the possibility of malignancy could not be ruled out from these findings, bilateral breast dynamic magnetic resonance imaging (MRI) was performed. MRI displayed a well-circumscribed mass with high signal intensity on T2-weighted images (T2WI) (Figure 3a). On contrast-enhanced T1-weighted images (T1WI), the mass showed strong enhancement with a microlobulated margin (Figure 3b). Dynamic contrast-enhanced imaging demonstrated rapid enhancement of the mass. The signal intensity peaked within 90 seconds after the intravenous injection of the contrast medium gadolinium (Figure 3c-f). Because the possibility of malignancy could also not be ruled out from these findings, core needle biopsy was performed. Histologically, the tumor was identified as fibroadenoma with focal mastopathic change. A lumpectomy was carried out under general anesthesia with a 10-mm margin to the adjacent breast tissue on the request of the patient 2 months later. Macroscopically, the mass was completely excised and the cut surface of the lesion showed a yellow-white, well-circumscribed, non-encapsulated mass, resembling a fibroadenoma (Figure 4).

**Figure 5:**
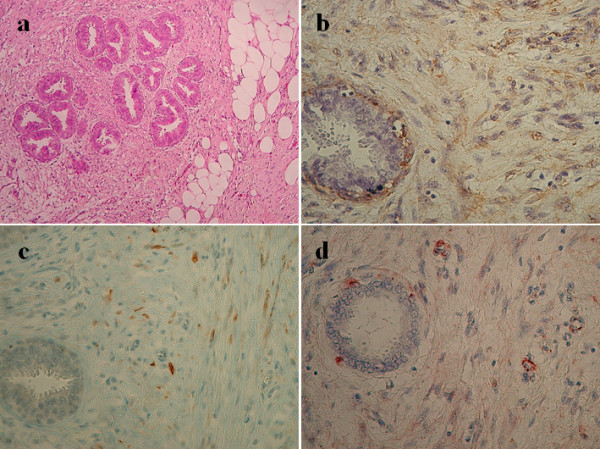
**Hematoxylin and eosin and immunohistochemical staining of the breast tumor specimen**. a) Hematoxylin and eosin staining of the breast tumor specimen shows a delicately encapsulated mass comprising fibrous and adipose tissue (Original magnification × 50). b-d) Immunohistochemical staining for smooth muscle actin, desmin, and HHF35 shows the smooth muscle histogenesis of stromal spindle cells (Original magnification × 150).

Microscopically, Hematoxylin and eosin staining showed benign ductal and lobular units, adipose tissue, fibrous stroma and bundles of elongated spindle cells in a disorganized pattern (Figure 5a). Immunohistochemically, the smooth muscle tissue stained diffusely positive for smooth-muscle actin (SMA), vimentin, desmin, and HHF35 (Figure 5b-d). In contrast, both cytokeratin and S-100 protein expressions in the stroma were negative.

## Discussion

Breast hamartomas comprise about 0.7-5% of all benign breast masses, and their main characteristic is the variety of tissue they contain [4]. Myoid hamartoma is a rare benign lesion of the breast, and is considered a variant of mammary hamartoma [5]. It was described for the first time in 1973 by Davies and Riddell [6]. According to some reports, this lesion is well-circumscribed and shows oval or round opacity on mammography, and ultrasonography reveals an internally iso- or hypoechoic, solid mass with regular margins [7,8]. Concerning mammography and ultrasonography, the findings of the present case are different from previous reports of myoid hamartoma.

On the other hand, few reports have described dynamic MRI findings for myoid hamartoma. Wong et al. reported that the signal intensity curve shows an early and significant rise on dynamic contrast enhancement [8]. Ko et al. revealed early intense enhancement and washout on dynamic MRI [9]. In the present case, the mass showed strong enhancement with a microlobulated margin on T1WI, and rapid enhancement on dynamic MRI, which differed from ordinary hamartoma [10].

Some reports have suggested that the diagnosis of myoid hamartoma might be wrong if only a few bioptic tissues could be taken, such as in core needle biopsy. They recommend open excisional biopsy to reach a more decisive diagnosis [3,11,12].

The histological differential diagnosis of myoid hamartoma should include various benign and malignant spindle cell tumors and tumor-like lesions. Lesions that should be considered include fibroadenoma, adenomyoepithelioma, leiomyosarcoma, and metaplastic breast carcinoma [13-15].

Immunohistochemical studies were helpful in making a diagnosis, and several reports have suggested that spindle and epithelioid tumor cells show strong positive staining for SMA, desmin, and vimentin, and the absence of staining for cytokeratin as well as S-100 protein [3,5,15,16]. In the present case, the stroma stained strongly for SMA, desmin, and HHF35, consistent with previous reports.

In this case, we followed the diagnosis according to core needle biopsy, which is very useful to differentiate between benign and malignant lesions. However, diagnosing a rare disease such as myoid hamartoma using the limited tissue yield of core needle biopsy is difficult [3,11,17]. In addition, the findings on mammography, ultrasonography, and dynamic MRI were different from the histological findings of core needle biopsy.

## Conclusion

In the diagnosis of myoid hamartoma, open excisional biopsy and immunohistochemical studies are crucial, and help to avoid confusion with various benign and malignant spindle cell tumors.

## Consent

Written informed consent was obtained from the patient for publication of this case report and accompanying images. A copy of the written consent is available for review by the Editor-in-Chief of this medical journal.

## Abbreviations

MRI: magnetic resonance imaging; T2WI: T2-weighted images; T1WI: T1-weighted images; SMA: smooth-muscle actin.

## Competing interests

The authors declare that they have no competing interests.

## Authors' contributions

MN drafted and co-wrote the manuscript with MM. SK, MM, NK, and SM were involved in the clinical care of the patient. IA, YS, and KE reported pathological findings and prepared slides for manuscript inclusion. GM evaluated the MRI findings of the breast mass. TT helped draft the final version of the manuscript. All authors have read and approved the final version of the manuscript.
